# Backstepping Sliding Mode Control for Radar Seeker Servo System Considering Guidance and Control System

**DOI:** 10.3390/s18092927

**Published:** 2018-09-03

**Authors:** Yexing Wang, Humin Lei, Jikun Ye, Xiangwei Bu

**Affiliations:** Air and Missile Defense College, Air Force Engineering University, Xi’an 710051, China; hmleinet@21cn.com (H.L.); jikunbo@sina.com (J.Y.); buxiangwei1987@126.com (X.B.)

**Keywords:** seeker servo system, guidance system, stabilized platform, backstepping sliding mode control, adaptive RBFNN, high-order tracking differentiator

## Abstract

This paper investigates the design of a missile seeker servo system combined with a guidance and control system. Firstly, a complete model containing a missile seeker servo system, missile guidance system, and missile control system (SGCS) was creatively proposed. Secondly, a designed high-order tracking differentiator (HTD) was used to estimate states of systems in real time, which guarantees the feasibility of the designed algorithm. To guarantee tracking precision and robustness, backstepping sliding-mode control was adopted. Aiming at the main problem of projectile motion disturbance, an adaptive radial basis function neural network (RBFNN) was proposed to compensate for disturbance. Adaptive RBFNN especially achieves online adjustment of residual error, which promotes estimation precision and eliminates the “chattering phenomenon”. The boundedness of all signals, including estimation error of high-order tracking differentiator, was especially proved via the Lyapunov stability theory, which is more rigorous. Finally, in considered scenarios, line of sight angle (LOSA)-tracking simulations were carried out to verify the tracking performance, and a Monte Carlo miss-distance simulation is presented to validate the effectiveness of the proposed method.

## 1. Introduction

A radar seeker is the “eye” of a missile, and it’s one of the most important parts of and the biggest sensor in the missile. The seeker guides the missile to attack a target because of its ability of detecting and tracking. A radar seeker servo system with platform (RSSSP) is a kind of high-precision servo tracking system, which is mounted on the front of a missile to achieve stable tracking of moving targets, while the object of the controlling system is an inertially stabilized platform (ISP). An ISP is widely used in varieties of seekers, including radar seekers [1] and infrared seekers; the application of ISP also includes aerial shooting, airborne remote sensing systems [2], robotics [3], deep-space exploration, etc. The main factors that affect the tracking performance are projectile motion interference, friction torque between the shafts, and the uncertainty of modelling [4,5]. Thus, ISP is a nonlinear, time-varying system with complex disturbance and parameter perturbation.

To realize a better dynamic response performance and stronger robustness of RSSSP, researchers have tried a variety of approaches. For simple control structure, the proportional-integral-derivative control has been used in many systems [6]; however, the contradiction between robustness and rapidity can’t be solved. Similarly, although $H_{\propto }$ control is introduced to eliminate the sensitivity for disturbance, the robustness conflicts with its performance more sharply [7,8]. Besides that, it has strong reservation towards control performance. Furthermore, sliding mode control (SMC) is also used to deal with the nonlinearity of ISP systems [9], as it enables all system states with arbitrary values to converge to a user-specified surface. Aiming at its shortcoming of the “chattering phenomenon”, many researchers applied high-order SMC; this method guarantees strong robustness as well as weaker chattering [10,11]. Furthermore, G.P. Incremona et al. designed an adaptive suboptimal second-order sliding mode control for microgrids [12]. The method considers the upper bound of uncertain terms unknown, which promotes the theory and application of SMC. Besides that, some researchers investigated model-free sliding mode control and acquired satisfying experimental results, which extended the application of SMC [13]. But, faced with compound disturbance of the ISP, using SMC separately is hard to guarantee control precision. SMC is usually combined with an estimation algorithm, which acquires better control performance [14].

Thus, the active disturbance rejection control technique is widely used in ISPs to compensate for disturbance [15,16], which eliminates disturbance on a large scale. Due to the compensation of the disturbance, the robustness and tracking precision of ISP are enhanced. In recent years, many intelligent algorithms are applied to ISPs. W. Yu applies a backstepping control method to control the optoeletronic system on the flexible suspend system, but the complex disturbance may eliminate the performance of system [17]. The neural network (NN) is used by many researchers to deal with uncertain nonlinear disturbance because of its universal approximation ability [18]. However, it requires training time and sample time to become NN-optimized, which is hard for RSSSP. In Reference [19,20], an adaptive RBFNN was proposed to generate the feedback control parameters online, while the extended state observer was used to compensate for composite disturbances. The control strategy possessed perfect adaptability and robustness. However, its control performance was easily affected by the selected upper bound of residual approximation error.

Especially few researchers combined the guidance and control systems to analyze seeker servo systems [21,22,23,24,25]; they mostly focus on improving the performance of servo systems separately by giving specific reference and disturbance signals, which may violate a realistic combat background.

To achieve high performance of RSSSP in a complete missile system, a model containing a guidance system, control system, and RSSSP is built, which contains unknown model perturbation and external disturbance. To guarantee the application of the designed algorithm, a designed high-order tracking differentiator (HTD) was used to estimate unknown system states. Based on the HTD, a backstepping sliding mode control was designed. Furthermore, an adaptive RBFNN was designed to compensate for complex disturbances, which would also eliminate the chattering problem. The special contributions of this paper are summarized as follows:

(1) Differently from existing studies, this paper combined the RSSSP with missile guidance and control systems to design a control algorithm, and a Monte Carlo simulation was carried out to verify the improvement of guidance precision, which is more realistic than analyzing servo systems by themselves;
(2) differently from traditional research in which the reference signal is given as a specific function, this paper applies HTD to estimate system states in real time, and all signals involved were generated in real time;
(3) different from traditional RBFNN, this paper proposed an adaptive RBFNN that adjusts the residual error in time, which enhances the estimation precision. No training is needed.

The paper is organized as follows: In Section 2, the model containing guidance, control, and RSSSP was built and its working preliminaries are presented. In Section 3, sliding backstepping controller was designed, along with the stability analysis. In Section 4, simulations were carried out to demonstrate effectiveness.

## 2. System Modeling and Problem Formulation

### 2.1. Constitution and Operating Principle of Two-Axis RSSSP

**Figure 1:**
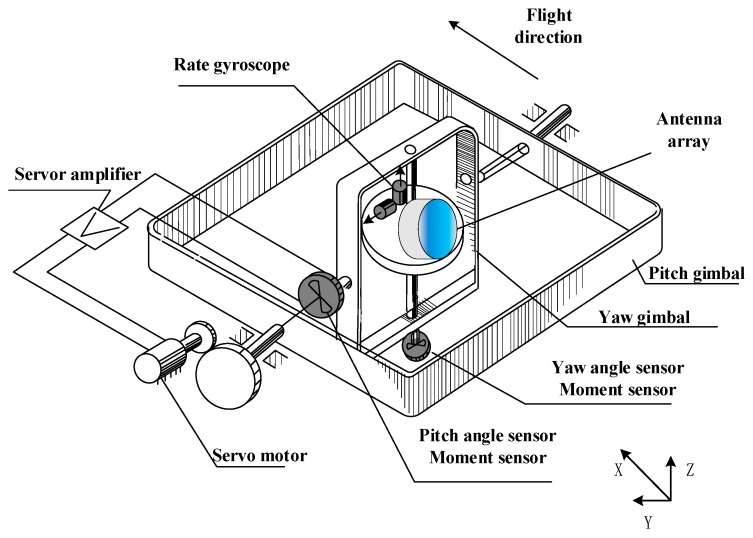
Schematic diagram of the two-axis radar seeker servo system with platform (RSSSP).

Figure 1 shows the schematic diagram of two-axis RSSSP. We can see that stabilized platform consists of two gimbals, which are pitch gimbal and yaw gimbal, respectively. The system is driven by two servo motors; the detective sensor was placed in the inner frame. The radar seeker antenna array is a high-precision compositive sensor that detects targets by emitting radio signals and receiving signals. The length and width of the pitch gimbal were 32.8 cm and 32.8 cm, respectively. While the length and width of yaw gimbal were 28.6 cm and 28.6 cm, respectively. The diameter of the antenna array was 25.0 cm.

From Figure 1, we can see the relationships between two gimbals: gyroscopes measuring the angular rate of the pitch and yaw gimbal, angle sensors measuring the angle of the pitch and yaw gimbal, and moment sensors measuring the moment of the pitch and yaw motor. Those sensors are the foundation of control system, which offer crucial feedback state information of the RSSSP.

ISP is fixed at the projectile body to achieve target angle alignment.

Because of the low coupling and similar characteristic of the pitch and yaw channel [5], we chose the pitch channel to analyze. Furthermore, the guidance system was analyzed in the longitudinal plane.

**Figure 2:**
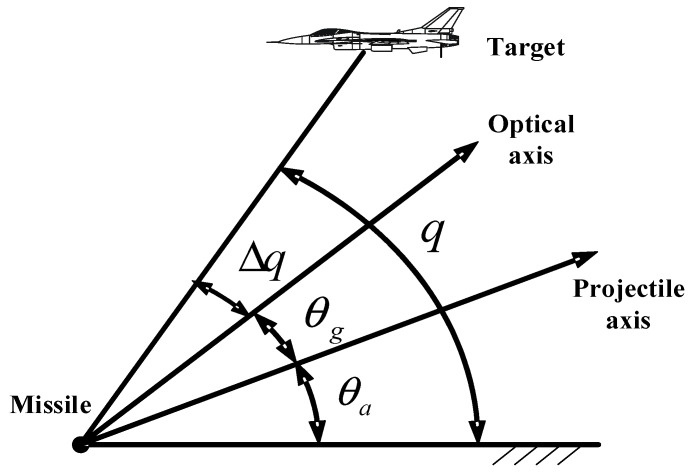
Angle relationship of radar seeker tracking.

Figure 2 shows the angle relationship of radar seeker tracking. q is the line of sight angle (LOSA), i.e., the angle between horizontal plane and connection of missile and target. The antenna array was trying to align at the target, while, because of the relative motion between missile and target, there unavoidably existed misalignment angle Δq, which should be eliminated. $\theta_{a}$ is the angle between the missile lengthwise axis and horizontal plane, while $\theta_{g}$ is the rotation angle of the antenna array surface in pitch channel.

To eliminate Δq quickly and accurately, a designed algorithm gave demand to the servo motor to drive the antenna array. In practice, $\theta_{g}$ plus $\theta_{a}$ is considered as the LOSA, and the changing rate of it is the LOSA rate, which is necessary for the guidance system.

### 2.2. Dynamic Model of the RSSSP

**Figure 3:**
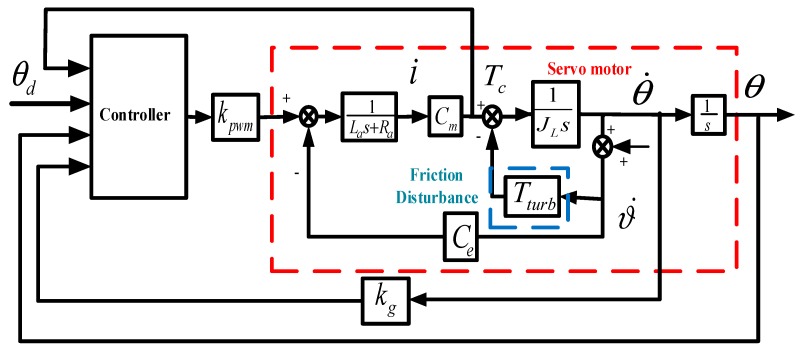
Block diagram of the RSSSP tracking loop.

Figure 3 shows the pitch channel block diagram of the RSSSP, where the block within the red imaginary line stands for the servo motor, while the block within the blue imaginary line stands for the friction disturbance; $\theta_{d}$ represents the angle conference signal of the system; $k_{PWM}$ is the power-amplifier coefficient; $\dot{\theta }$ is the angular rate of stabilized platform in inertial space; $\dot{\vartheta }$ is the projectile pitch angle rate; $T_{turb}$ is the disturbance moment; $k_{g}$ is the simplified transfer function of rate gyroscope; $T_{c}$ is the moment output of servo motor; i is the electric current of the servo motor; $J_{L}$ is the rotational inertia of motor load; $L_{a}$ is the inductance of inductance of armature winding; $R_{a}$ is the resistance of armature winding, $C_{m}$ is the moment coefficient of motor; $C_{e}$ is the coefficient of counter electromotive force.

Combined with the dynamic equation of the stable platform and the dynamic equation of the motor, the mathematical model of the RSSSP is acquired as follows [5]:

(1) $$\left\{ \begin{array}{l} {\dot{x}}_{1}=x_{2}\\ {\dot{x}}_{2}=\frac{x_{3}}{J_{L}}-\frac{T_{turb}}{J_{L}}\\ {\dot{x}}_{3}=-\frac{C_{m}C_{e}}{L_{a}}x_{2}-\frac{R_{a}}{L_{a}}x_{3}+\frac{C_{m}k_{PWM}}{L_{a}}u-\frac{C_{e}C_{m}\Delta }{L_{a}} \end{array}\right.$$

where $x_{1},x_{2}\begin{array}{c} ,x_{3} \end{array}$ are state variables, which represent $\theta ,\dot{\theta }$ and $T_{c}$, respectively u is the output of controller.

The disturbance torque mainly consists of the spring torque and damping torque. The spring torque mainly results from the drag of wire when the platform is rotating towards the projectile, while the damping torque mainly results from the interaction between platform and base. The torque model is shown as Equation (2), where $K_{w}$ and $K_{N}$ are the proportionality of the spring torque and damp torque, respectively [21]:

(2) $$T_{turb}=K_{W}\dot{\theta }+K_{N}\theta$$

### 2.3. Dynamic Model of Guidance and Control Systems

**Figure 4:**
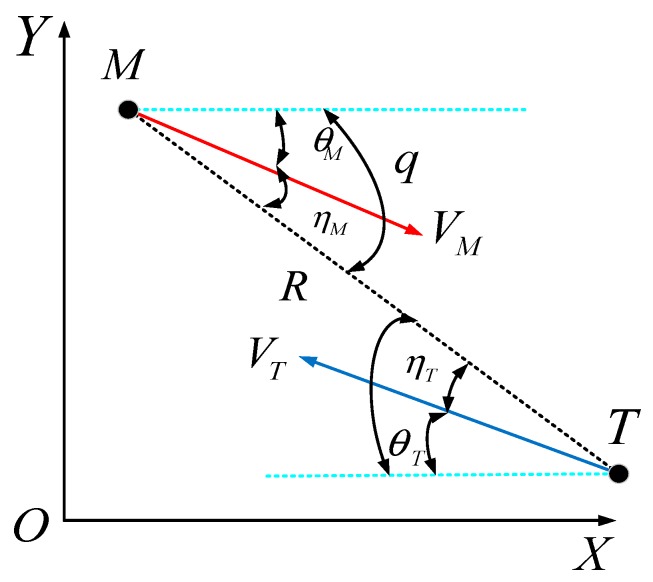
Diagram of missile-target motion relationship.

Figure 4 demonstrates the relationship of missile–target motion, where $V_{M},V_{T}$ represent the velocity of missile and target, respectively, $\theta_{m},\theta_{T}$ are the trajectory inclination angular of missile and target, respectively, and R is the distance between missile and target. The relative motion equation between missile and target is given as follows:

(3) $$\dot{R}=-V_{M}\cos\eta_{M}-V_{T}\cos\eta_{T}$$

(4) $$R\dot{q}=V_{M}\sin\eta_{M}+V_{T}\sin\eta_{T}$$

(5) $${\dot{\theta }}_{M}=A_{M}/V_{M}$$

(6) $${\dot{\theta }}_{T}=A_{T}/V_{T}$$

where $\theta_{M}=q-\eta_{M},\theta_{T}=q-\eta_{T}$, and $A_{M},A_{T}$ represent the command acceleration of missile and target, respectively [26].

The simplified missile dynamics and autopilot model are expressed as $\frac{\left(T_{\alpha }s+1\right)}{V_{m}},\frac{1}{{\left(T_{Auto}s+1\right)}^{3}}$, respectively. $T_{\alpha },T_{Auto}$ are the turning rate time constant of missile maneuverability and the time constant of autopilot, respectively.

**Figure 5:**
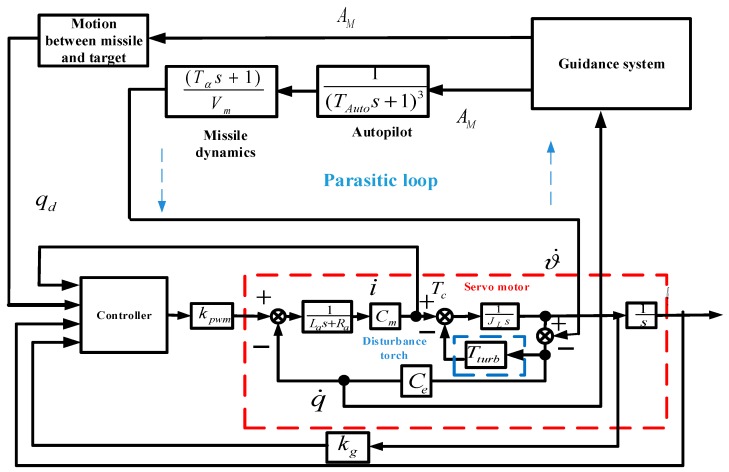
Complete model of missile control system (SGCS).

Above all, a complete model is established in Figure 5.

**Remark** **1.** *Through the complete model in Figure 5, we can connect the servo system to the guidance system; furthermore, the motion between missile and target will be acquired (as seen in Figures 8b and 9b). Different from traditional research on RSSSP, the reference tracking signal comes from an angle signal detected by the detective sensor (seen in Figures 8a, 9a and 12) in real time. The projectile attitude motion generated by the missile control system is also regarded as disturbance* $\dot{\vartheta }$ *(as seen in Figures 10 and 13), which can test the disturbance-isolation ability of RSSSP. The working situation of RSSSP is close to real combat situations. Moreover, a Monte Carlo target miss-distance simulation (seen in Table 2) is accessible via the established model.*

**Remark** **2.** *The model describes the complete system relative to seeker servo systems except for signal-processing systems. Considering that the signal-processing system is independent of controller design, the system is ignored in modeling.*

### 2.4. Control Problems for RSSSP

There are some troublesome characteristics in the RSSSP:

(1) When there exist torque disturbances and angular-rate disturbances generated by projectile motion, high-performance angle tracking is hard to guarantee;
(2) due to the change of the flight environment and the limited accuracy of mathematical modeling, coefficient uncertainty, and perturbation, tracking performance may not be guaranteed;
(3) to enhance the performance of RSSSP, the states have to be estimated precisely in real time.

## 3. Controller Design

**Figure 6:**
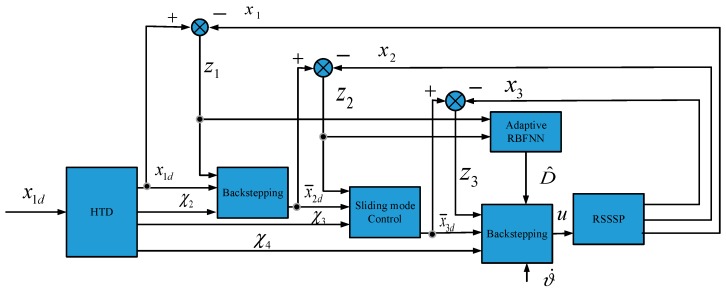
Control block diagram of proposed method.

The control block diagram of the proposed method is shown in Figure 6.

To enhance tracking precision and robustness, a sliding mode backstepping control based on an adaptive RBFNN is proposed. The proposed method was designed under the structure of backstepping control. The system can be considered as a three-loop control system, where $x_{1d}$ is the reference input of system, ${\bar{x}}_{2d}$ can be considered as the first virtual control term for angular velocity control loop, ${\bar{x}}_{3d}$ is the virtual control term for torque loop, u is the actual control term. To ensure the feasibility of the designed algorithm, newly defined states $\chi_{2},\chi_{3},\chi_{4}$ are essential, which will be estimated by designed HTD. $\dot{\vartheta }$ is the motion disturbance generated by the missile control system. Aiming at the problem of compound disturbance, an adaptive RBFNN was applied. $\hat{D}$ is the transferred disturbance estimated online, which would be used to offset disturbance.

Compared with traditional designs of sliding mode control, this paper applies sliding mode control only in an angular velocity loop to enhance the robustness of angular velocity tracking. To avoid a severe “chattering phenomenon”, sliding mode control is not used in angular and torque tracking loops. The upper bound of residual error is also estimated online to reduce the “chattering phenomenon”.

In this section, high-order tracking differentiator and adaptive RBFNN were designed at first, and were then used in controller design in Section 3.3. Sliding mode backstepping control based on adaptive RBFNN was designed for better performance of RSSSP. Furthermore, stability analysis was carried out in Section 3.4, and the semiglobal uniform ultimate stability of the system was proved.

### 3.1. High-Order Tracking Differentiator

In the subsequent developments, an HTD was designed to estimate newly-defined states. The HTD was formulated as follows:

(7) $$\begin{array}{l} {\dot{\chi }}_{1}=\chi_{2}\\ {\dot{\chi }}_{2}=\chi_{3}\\ {\dot{\chi }}_{3}=\chi_{4}\\ {\dot{\chi }}_{4}=R^{3}\left[-a_{1}\tanh(\chi_{1}-x(t))-a_{2}\tanh(\frac{\chi_{2}}{R})-a_{3}\tanh(\frac{\chi_{3}}{R^{2}})-a_{4}\tanh(\frac{\chi_{4}}{R^{3}})\right] \end{array}$$

where $R>0,a_{1}>0,a_{2}>0,a_{3}>0,a_{4}>0$ are positive constants to be chosen; x(t) is the input signal; $\chi_{1},\chi_{2},\chi_{3},\chi_{4}$ donate the states of HTD, which are $x(t),\dot{x}(t),\ddot{x}(t),\dddot{x}(t)$ respectively. The corresponding estimation errors are defined as follows: $e_{1}=\chi_{1}-x(t),e_{2}=\chi_{2}-\dot{x}(t),$ $e_{3}=\chi_{3}-\ddot{x}(t),e_{4}=\chi_{4}-\dddot{x}(t)$, by choosing an infinitely R for the HTD, we can get:

(8) $$\begin{array}{l} \underset{R\to +\infty }{\lim}(\chi_{1}-x(t))=\underset{R\to \infty }{\lim}e_{1}=0,\\ \frac{d\underset{R\to +\infty }{\lim}(\chi_{1}-x(t))}{dt}=\underset{R\to \infty }{\lim}({\dot{\chi }}_{1}-\dot{x}(t))=\underset{R\to \infty }{\lim}(\chi_{2}-\dot{x}(t))=\underset{R\to \infty }{\lim}e_{2}=0,\\ \frac{d\underset{R\to +\infty }{\lim}(\chi_{2}-\dot{x}(t))}{dt}=\underset{R\to \infty }{\lim}({\dot{\chi }}_{2}-\ddot{x}(t))=\underset{R\to \infty }{\lim}(\chi_{3}-\ddot{x}(t))=\underset{R\to \infty }{\lim}e_{3}=0,\\ \frac{d\underset{R\to +\infty }{\lim}(\chi_{3}-\ddot{x}(t))}{dt}=\underset{R\to \infty }{\lim}({\dot{\chi }}_{3}-\dddot{x}(t))=\underset{R\to \infty }{\lim}(\chi_{4}-\dddot{x}(t))=\underset{R\to \infty }{\lim}e_{4}=0. \end{array}$$

From the above deduction, the estimation errors $e_{i}(i=1,2,3,4)$ can converge to zero by choosing an adequately large R. If we choose an infinitely large but bounded R for HTD, there exist positive constants ${\bar{e}}_{i}(i=1,2,3,4)$, such that ${\bar{e}}_{i}\geq \left|e_{i}\right|\;(i=1,2,3,4)$ [27,28].

**Figure 7:**
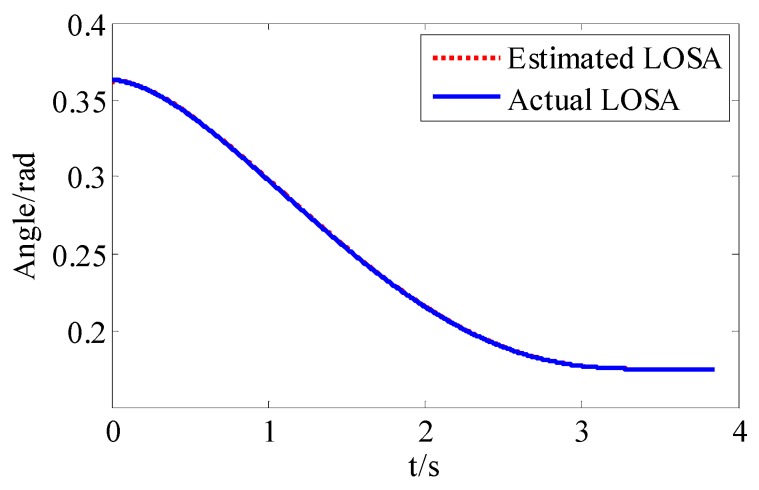
High-order tracking differentiator (HTD) estimation.

The application of HTD can be found in Figure 6, and the validation of HTD is verified in Figure 7.

### 3.2. Adaptive Neural Network

To guarantee the controller’s robustness, an adaptive RBFNN is introduced to approximate the compound disturbance owing to its excellent performance and global approximation [29]. The adaptive RBFNN is defined as the mapping relationship between input vector $X={\left[x_{1},x_{2},\ldots ,x_{n}\right]}^{T}\in R^{n}$ and the output y∈R [30].

(9) $$y=W^{T}h(X)$$

where $W={\left[w_{1},w_{2},\ldots w_{m}\right]}^{T}\in R^{m}$ donates weight vector; m and n represent the node number and input number, respectively; and $h(X)={\left[h_{1}(X),h_{2}(X),\ldots h_{m}(X)\right]}^{T}\in R^{m}$ with $h_{j}(X)$ is defined as follows:

(10) $$h_{j}(X)=\exp\left(-\frac{\left\|X-c\right\|}{\sigma_{j}^{2}}\right)\in R^{m},j=1,2,\ldots m$$

where $c={\left[c_{1},c_{2},\ldots ,c_{n}\right]}^{T}\in R^{m}$ and $\sigma ={\left[\sigma_{j1},\sigma_{j2},\ldots ,\sigma_{jn}\right]}^{T}\in R^{n}$ mean a center and a width vector of $h_{j}(X)$, respectively [31].

For an arbitrary continuous unknown function F(X), it has to be proven that there exists an ideal weight vector $W={\left[w_{1},w_{2},\ldots w_{m}\right]}^{T}\in R^{m}$, such that

(11) $$F(X)=W^{*}h(X)+\varepsilon$$

where ε is approximate error. It should be noted that $W^{*}$ and ε are unknown; their elements $w_{1}{}^{*},w_{2}{}^{*},\ldots w_{m}{}^{*}$, and ε are required to be adjusted adaptively.

Define the error between the ideal weight $W^{*}$ and the estimated weight $\hat{W}$ as

(12) $$\tilde{W}=\hat{W}-W^{*}$$

$\hat{\varepsilon }$ is the estimated value of ε. The adaptation laws of $\hat{W}$ and $\hat{\varepsilon }$ are designed in next Section.

The application of adaptive RBFNN can be found in Figure 6.

### 3.3. Controller Design for RSSSP

**Assumption** **1.** *We assume that the LOSA* $q_{d}$ *is detected accurately.*

**Assumption** **2.** *The reference signal* $x_{1d}$ *, its derivative* ${\dot{x}}_{1d}$ *, its second-order derivation* ${\ddot{x}}_{1d}$ *, and its third-order derivation* ${\dddot{x}}_{1d}$ *are limited.*

The algorithm design is taken as follows:

Define the state error as

(13) $$\begin{array}{l} z_{1}=x_{1}-x_{1d}\\ z_{2}=x_{2}-x_{2d} \end{array}$$

The time derivative of $z_{1}$ is obtained by

(14) $${\dot{z}}_{1}={\dot{x}}_{1}-{\dot{x}}_{1d}=x_{2}-{\dot{x}}_{1d}$$

Define the virtual control law as

(15) $$x_{2d}=-k_{1}z_{1}+{\dot{x}}_{1d}$$

Use $\chi_{2}$ to replace $x_{1d}$, Equation (15) becomes

(16) $${\bar{x}}_{2d}=-k_{1}z_{1}+\chi_{2}$$

where ${\bar{x}}_{2d}$ is available virtual control law owing to tracking differentiator, while ${\bar{x}}_{3d}$ in Equation (28) has the same meaning. Taking the derivative of $x_{2d}$, we can get

(17) $$\begin{array}{l} {\dot{x}}_{2d}=-k_{1}{\dot{z}}_{1}+{\ddot{x}}_{1d}=-k_{1}x_{2}+k_{1}{\dot{x}}_{1d}+{\ddot{x}}_{1d}\\ {\ddot{x}}_{2d}=-k_{1}{\ddot{z}}_{1}+{\dddot{x}}_{1d}=-k_{1}\frac{x_{3}}{J_{L}}+k_{1}\frac{T_{turb}}{J_{L}}+k_{1}{\ddot{x}}_{1d}+{\dddot{x}}_{1d} \end{array}$$

where $k_{1}$ is a positive constant value. Considering that $T_{turb}$ is a positive constant value. Considering that can’t be acquired directly, the item is ignored, and will be estimated later. Substituting $\chi_{3},\chi_{4}$ for ${\ddot{x}}_{1d},{\dddot{x}}_{1d}$, we can get:

(18) $$\begin{array}{l} {\bar{\dot{x}}}_{2d}=-k_{1}{\dot{z}}_{1}+{\ddot{x}}_{1d}=-k_{1}x_{2}+k_{1}\chi_{2}+\chi_{3}\\ {\bar{\ddot{x}}}_{2d}=-k_{1}{\ddot{z}}_{1}+{\dddot{x}}_{1d}=-k_{1}\frac{x_{3}}{J_{L}}+k_{1}\chi_{3}+\chi_{4} \end{array}$$

**Remark** **3.** *Considering that* ${\dot{x}}_{2d},{\ddot{x}}_{2d}$ *can’t be directly acquired, intermediate variables* ${\bar{\dot{x}}}_{2d},{\bar{\ddot{x}}}_{2d}$ *are defined, and will be used for controller design later.*

The first Lyapunov function is chosen as

(19) $$V_{1}=\frac{1}{2}{z_{1}}^{2}$$

Differentiating $V_{1}$ with respect to time, and we can get

(20) $$\begin{array}{lll} {\dot{V}}_{1} & = & z_{1}{\dot{z}}_{1}=z_{1}(x_{2}-{\dot{x}}_{1d\;})=z_{1}(x_{2}-x_{2d}-e_{2}-k_{1}z_{1})\\ & = & z_{1}(z_{2}-e_{2})-k_{1}z_{1}{}^{2} \end{array}$$

To enable that the LOSA rate tracking possesses strong robustness, sliding mode control is adopted to eliminate the effect of uncertain parameters perturbance.

Define a traditional sliding mode and a complementary sliding mode as follows:

(21) $$s=z_{2}+k_{2}\int z_{2}d\tau$$

(22) $$s_{c}=z_{2}-k_{2}\int z_{2}d\tau$$

where $k_{2}>0$ is the parameter to be designed.

And the second Lyapunov function is chosen as

(23) $$V_{2}=\frac{1}{2}s^{2}+\frac{1}{2}s_{c}{}^{2}$$

Take time derivative of Equation (21), and the following equation is acquired

(24) $$\dot{s}={\dot{z}}_{2}+k_{2}z_{2}=\frac{x_{3}-T_{turb}}{J_{L}}-{\dot{x}}_{2d}+k_{2}z_{2}$$

The relationship between s and $s_{c}$ can be expressed as

(25) $${\dot{s}}_{c}+k_{2}(s+s_{c})=\dot{s}$$

Then the virtual law is designed as

(26) $$x_{3d}=T_{turb}+J_{L}({\dot{x}}_{2d}-k_{2}z_{2}-k_{2}s)$$

Furthermore, we can get the derivative that will be used later

(27) $${\dot{x}}_{3d}={\dot{T}}_{turb}+J_{L}({\ddot{x}}_{2d}-k_{2}{\dot{z}}_{2}-k_{2}\dot{s})$$

Considering that $T_{turb}$ is an unknown function, $x_{3d}$ can’t be used directly; therefore, virtual control law is chosen as

(28) $${\bar{x}}_{3d}=J_{L}({\bar{\dot{x}}}_{2d}-k_{2}z_{2}-k_{2}s)$$

Besides, the derivation of $x_{3d}$ is

(29) $${\dot{x}}_{3d}={\dot{T}}_{turb}+J_{L}({\ddot{x}}_{2d}-k_{2}{\dot{z}}_{2}-k_{2}\dot{s})$$

Invoking Equations (16) and (21), the available virtual controller ${\bar{\dot{x}}}_{3d}$ is acquired

(30) $${\bar{\dot{x}}}_{3d}=J_{L}({\bar{\ddot{x}}}_{2d}-k_{2}(-\frac{2R_{a}}{L_{a}}x_{3}-(\frac{2C_{m}C_{e}}{L_{a}}+1)x_{2}))$$

To guarantee the convergence of $z_{3}$, the third Lyapunov is chosen as

(31) $$V_{3}=\frac{1}{2}z_{3}{}^{2}$$

Taking the derivation of $z_{3}$, we get the following equation:

(32) $$\begin{array}{lll} {\dot{z}}_{3} & = & {\dot{x}}_{3}-{\dot{x}}_{3d}\\ & = & -\frac{R_{a}}{L_{a}}x_{3}-\frac{C_{m}C_{e}}{L_{a}}x_{2}-\frac{C_{e}C_{m}\dot{\vartheta }}{L_{a}}+\frac{C_{m}k_{PWM}}{L_{a}}u-{\dot{x}}_{3d} \end{array}$$

Design the actual control law as follows

(33) $$u=\frac{L_{a}}{C_{m}k_{PWM}}(\frac{R_{a}}{L_{a}}x_{3}+\frac{C_{m}C_{e}}{L_{a}}x_{2}-{\bar{\dot{x}}}_{3d}-z_{3})-D$$

where the whole disturbance D is presented as

(34) $$D=\frac{L_{a}}{C_{m}k_{PWM}}(\frac{z_{2}}{z_{3}}(s+s_{c}+2k_{1})+(T_{turb}+(2-2k_{2}J_{L}){\dot{T}}_{turb}+\frac{C_{e}C_{m}\dot{\vartheta }}{L_{a}})/z_{3})$$

Considering that D is a complex function with $z_{1},z_{2},z_{3}$ as arguments, an adaptive RBFNN is introduced to approximate D on line.

**Remark** **4.** *In Equation (35),* $T_{turb}$ *and* ${\dot{T}}_{turb}$ *are functions with* $z_{1},z_{2}$ *as arguments. Besides, the projectile attitude motion disturbance* $\dot{\vartheta }$ *is seduced by guidance command, which can be written as* $\dot{\vartheta }(a_{n})$ *, while guidance command is determined by the LOSA rate, which can be seen as* $a_{n}(z_{1},z_{2},z_{3})$ *. Above all,* $\dot{\vartheta }$ *can be seen as the function with* $z_{1},z_{2},z_{3}$ *as arguments, which can be rewritten as* $\dot{\vartheta }(a_{n}(z_{1},z_{2},z_{3}))$ *. Therefore,* $\dot{\vartheta }$ *can be estimated by RBFNN.*

Therefore, D can be expressed as

(35) $$D=W^{*}h(z)+\varepsilon_{1}$$

where $z={\left[z_{1}\begin{array}{c} \begin{array}{c} {\begin{array}{cc} & z \end{array}}_{2}{\begin{array}{cc} & z \end{array}}_{3} \end{array} \end{array}\right]}^{T}$.

The control law is rewritten as

(36) $$u=\frac{L_{a}}{C_{m}k_{PWM}}(\frac{R_{a}}{L_{a}}x_{3}+\frac{C_{m}C_{e}}{L_{a}}x_{2}-{\bar{\dot{x}}}_{3d}-z_{3})-\hat{W}h(z)-{\hat{\varepsilon }}_{1}$$

To develop the adaption laws of $\tilde{W}$ and $\hat{\varepsilon }$, define Lyapunov function

(37) $$V_{4}=V_{1}+V_{2}+V_{3}+\frac{1}{2\gamma_{1}}{\tilde{W}}^{T}\tilde{W}+\frac{1}{2\gamma_{2}}{\left({\hat{\varepsilon }}_{1}-\varepsilon_{1}\right)}^{2}$$

Combining with Equations (20), (28), (30) and (31), the derivation of $V_{4}$ is acquired.

(38) $$\begin{array}{ll} {\dot{V}}_{4} & ={\dot{V}}_{1}+{\dot{V}}_{2}+{\dot{V}}_{3}+\frac{1}{\gamma_{1}}{\tilde{W}}^{T}\dot{\tilde{W}}+\frac{1}{\gamma_{2}}({\hat{\varepsilon }}_{1}-\varepsilon_{1}){\dot{\hat{\varepsilon }}}_{1}\\ & =z_{1}(z_{2}-e_{2})-k_{1}z_{1}{}^{2}+(s+s_{c})(-k_{1}e_{2}+e_{3})-k_{3}z_{3}{}^{2}\\ & +z_{3}((J_{L}k_{3}+1)(k_{1}e_{2}+e_{3})+J_{L}k_{3}(k_{1}e_{3}+e_{4})+D-\hat{W}h(z)-{\hat{\varepsilon }}_{1})\\ & -k_{2}{\left(s+s_{c}\right)}^{2}+\frac{1}{\gamma_{1}}{\tilde{W}}^{T}\dot{\tilde{W}}+\frac{1}{\gamma_{2}}({\hat{\varepsilon }}_{1}-\varepsilon_{1}){\dot{\hat{\varepsilon }}}_{1} \end{array}$$

Since $W^{*}$ is a constant, it should be noted that $\dot{\tilde{W}}=\dot{\hat{W}}$.

According to Equation (38), adaption laws $\dot{\hat{W}}$ and ${\dot{\hat{\varepsilon }}}_{1}$ are designed as follows:

(39) $${\dot{\hat{\varepsilon }}}_{1}=\gamma_{2}z_{2}$$

(40) $$\dot{\hat{W}}=\gamma_{1}z_{2}h(z)$$

### 3.4. Stability Analysis

**Theorem** **1.** *Consider the closed-loop system Equation (1) and controller Equation (36), adaptive laws Equations (39) and (40), all the signals involved in Equation (37) are bounded.*

**Proof.**  Substituting Equations (20), (24), (25), (39) and (40) into Equation (38), we get

(41) $$\begin{array}{ll} {\dot{V}}_{4} & ={\dot{V}}_{1}+{\dot{V}}_{2}+{\dot{V}}_{3}+\frac{1}{\gamma_{1}\;}{\tilde{W}}^{T}\dot{\tilde{W}}+\frac{1}{\gamma_{2}}({\hat{\varepsilon }}_{1}-\varepsilon_{1}){\dot{\hat{\varepsilon }}}_{1}\\ & =z_{1}(z_{2}-e_{2})-k_{1}z_{1}{}^{2}+(s+s_{c})(-k_{1}e_{2}+e_{3})-k_{3}z_{3}{}^{2}\\ & +z_{3}((J_{L}k_{3}+1)(k_{1}e_{2}+e_{3})+J_{L}k_{3}(k_{1}e_{3}+e_{4}))-2k_{2}z_{2}{}^{2} \end{array}$$

☐

**Remark** **5.** *From Equation (41), it can be observed that the global boundedness of disturbance is guaranteed. Besides, the chattering phenomenon caused by sliding mode control is eliminated.*

Notice that

(42) $$\left\{ \begin{array}{l} z_{1}z_{2}-z_{1}e_{2}+2z_{2}(-k_{1}e_{2}+e_{3})\\ \leq \frac{z_{1}{\;}^{2}+z_{2}{}^{2}}{2}+{\bar{e}}_{2}(\frac{1}{2}+\frac{z_{1}{}^{2}}{2})+\sqrt{k_{1}{}^{2}{\bar{e}}_{2}{}^{2}+{\bar{e}}_{3}{}^{2}}(1+z_{2}{}^{2})\\ z_{3}((J_{L}k_{3}+1)(k_{1}{\bar{e}}_{2}+{\bar{e}}_{3})+J_{L}k_{3}(k_{1}{\bar{e}}_{3}+{\bar{e}}_{4}))\\ \leq B_{1}(\frac{1}{2}+\frac{z_{3}{}^{2}}{2}) \end{array}\right.$$

where $B_{1}=\sqrt{{\left(J_{L}k_{3}+1\right)}^{2}(k_{1}{\bar{e}}_{2}{}^{2}+{\bar{e}}_{3}{}^{2})+J_{L}{}^{2}k_{3}{}^{2}(k_{1}{}^{2}{\bar{e}}_{3}{}^{2}+{\bar{e}}_{4}{}^{2})}$.

Thus, the following inequality is acquired.

(43) $$\begin{array}{lll} {\dot{V}}_{4} & \leq & \frac{z_{1}{}^{2}+z_{2}{}^{2}}{2}+{\bar{e}}_{2}(\frac{1}{2}+\frac{z_{1}{}^{2}}{2})+\sqrt{k_{1}{}^{2}{\bar{e}}_{2}{}^{2}+{\bar{e}}_{3}{}^{2}}(1+z_{2}{}^{2})+B_{1}(\frac{1}{2}+\frac{z_{3}{}^{2}}{2})\\ & = & (\frac{{\bar{e}}_{2}}{2}+\frac{1}{2}-k_{1})z_{1}{}^{2}+(\frac{1}{2}+\sqrt{k_{1}{}^{2}{\bar{e}}_{2}{}^{2}+{\bar{e}}_{3}{}^{2}}-k_{2})z_{2}{}^{2}+(\frac{B_{1}}{2}-k_{3})z_{3}{}^{2}+B_{2} \end{array}$$

where $B_{2}=\frac{1}{2}({\bar{e}}_{2}+\sqrt{k_{1}{}^{2}{\bar{e}}_{2}{}^{2}+{\bar{e}}_{3}{}^{2}}+B_{1})$.

Let

(44) $$k_{1}>\frac{{\bar{e}}_{2}}{2}+\frac{1}{2},k_{2}>\frac{1}{2}+\sqrt{k_{1}{}^{2}{\bar{e}}_{2}{}^{2}+{\bar{e}}_{3}{}^{2}},k_{3}>\frac{B_{1}}{2}$$

and define the following compact sets:

(45) $$\begin{array}{l} \Omega_{z_{1}}=\left\{z_{1}|\left|z_{1}\right|\leq \sqrt{\frac{B_{2}}{\frac{{\bar{e}}_{2}}{2}+\frac{1}{2}-k_{1}}}\right\}\\ \Omega_{z_{2}}=\left\{z_{2}|\left|z_{2}\right|\leq \sqrt{\frac{B_{2}}{\frac{1}{2}+\sqrt{k_{1}{}^{2}{\bar{e}}_{2}{}^{2}+{\bar{e}}_{3}{}^{2}}-k_{2}}}\right\}\\ \Omega_{z_{3}}=\left\{z_{3}|\left|z_{3}\right|\leq \sqrt{\frac{B_{2}}{\frac{B_{1}}{2}-k_{3}}}\right\} \end{array}$$

It can be seen that ${\dot{V}}_{4}$ will be negative if $z_{1}\notin \Omega_{z_{1}}$ or $z_{2}\notin \Omega_{z_{2}}$ or $z_{3}\notin \Omega_{z_{3}}$. Hence, $z_{1},z_{2},z_{3}$ are semiglobally uniformly ultimately bounded. This is the end of proof.

## 4. Simulation and Analysis

Simulations were carried out in this section to verify the effectiveness of the proposed control scheme. We consider two different combat scenarios to verify the algorithm. The designed algorithm was applied to the missile RSSSP. In the first scenario, the missile attacked the target from upwards. In the second scenario, the missile attacked the target from downwards and the target was carrying out sine maneuvering.

The parameters of the RSSSP are shown in Table 1.

*Case 1.* In this case, we assume that a missile intercepts a low-altitude target. The positions of missile and target are (0 m,2000 m) and (5000 m,100 m), respectively; their speeds are 1000 m/s and (400+30×t)m/s, respectively. Thus, the LOSA is 0.36 rad, and the initial servo system rotation angle was 0.36 rad, while the initial trajectory inclination angles were 30° and 0°, respectively. Because of the multipath effect of a radar seeker while interpreting low-altitude targets, Brewster angle restraint has to be guaranteed, and the double sliding mode guidance law [32] is designed as

(46) $$\begin{array}{l} A_{M}=(-2\dot{R}\dot{q}+\lambda V_{M}S_{2}-k\dot{R}S-\lambda V_{M}\dot{R}S_{1}/R\\ -\cos\eta_{T}A_{T}+\varepsilon sgn(S(t))/\cos\eta_{m} \end{array}$$

where λ,k,ε are positive design parameters, $S_{1}=x_{1}-x_{1d}$, $S_{2}=x_{2}$, $S=S_{2}+\lambda V_{M}S_{1}/R$.

The coefficients of the missile control system are $T_{\alpha }=1.5$, $T_{Auto}=0.06$, respectively.

The order of HTD is chosen as 2, and the design parameters of HTD are chosen as follows: $R_{1}=200,R_{2}=20,R_{3}=5$, $a_{11}=10,a_{21}=10,a_{31}=10,a_{12}=10,a_{22}=10,a_{31}=10$; the control design parameters are $k_{1}=10,k_{2}=3,k_{3}=0.0125$.

Parameters of RBFNN are chosen as $\gamma_{1}=1000,\gamma_{2}=3$, the number of node is 100, and the center of RBFNN is evenly spaced in c∈[−50,49], the width is chosen as b=15. In particular, the mentioned parameters are all designed, while $\hat{W}$ and $\hat{\varepsilon }$ are adaptive vector and adaptive parameter that adjust online.

The effectiveness of HTD is shown as follows; it can be observed from Figure 16 that the estimation of LOSA is perfect.

To show the priority, the proposed method is compared with a prescribed performance controller in Reference [20].

**Figure 8:**
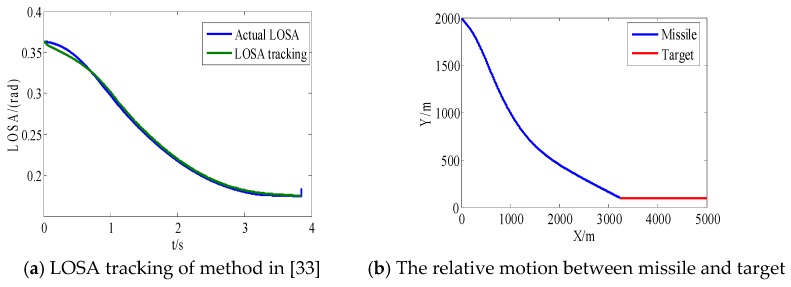
Method in Reference [33].

**Figure 9:**
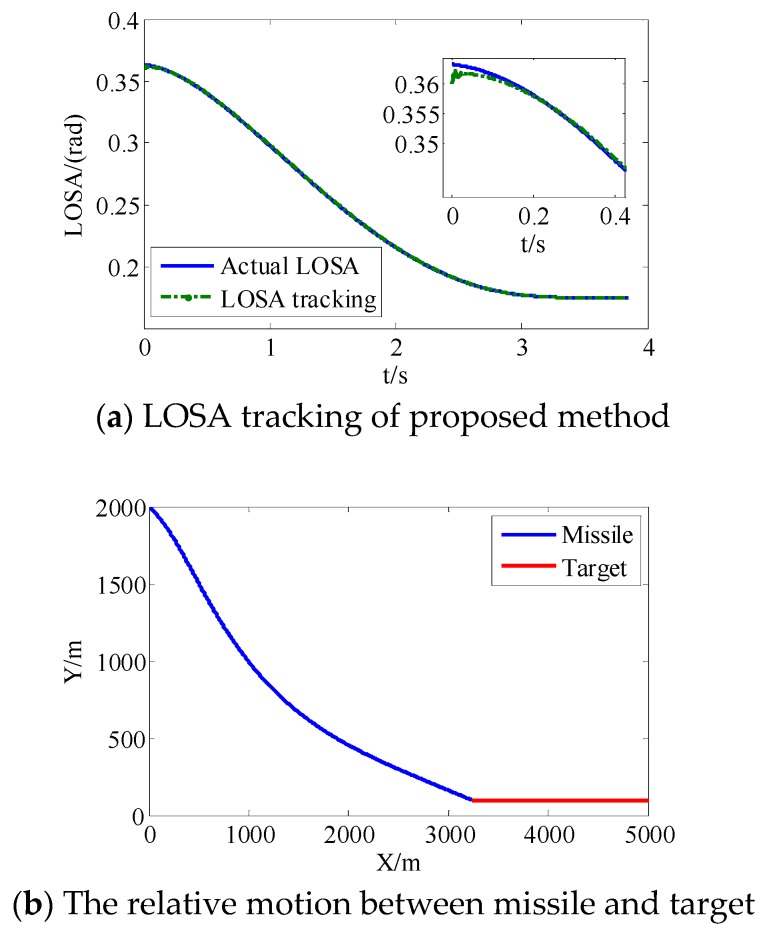
Proposed method.

**Figure 10:**
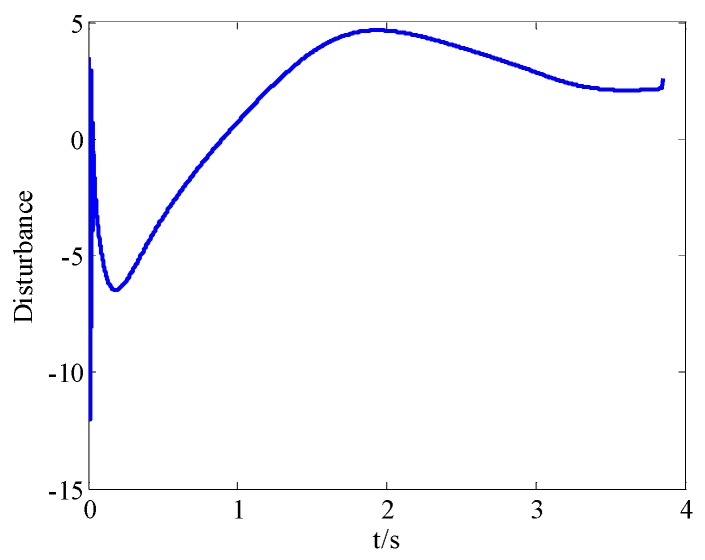
Projectile disturbance in real time.

**Figure 11:**
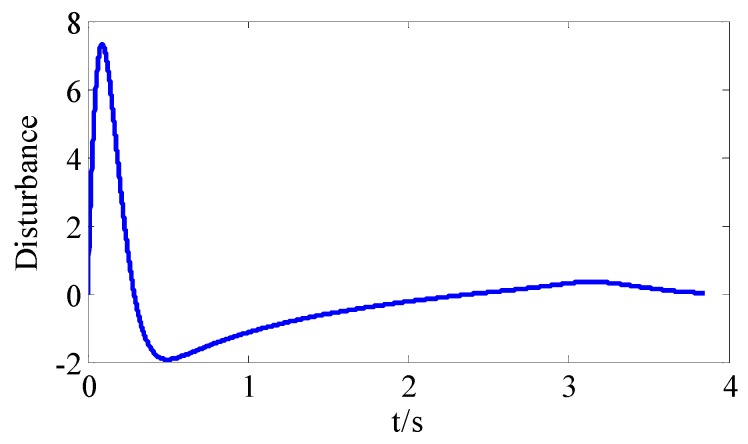
Total disturbance estimated by adaptive RBFNN.

**Figure 12:**
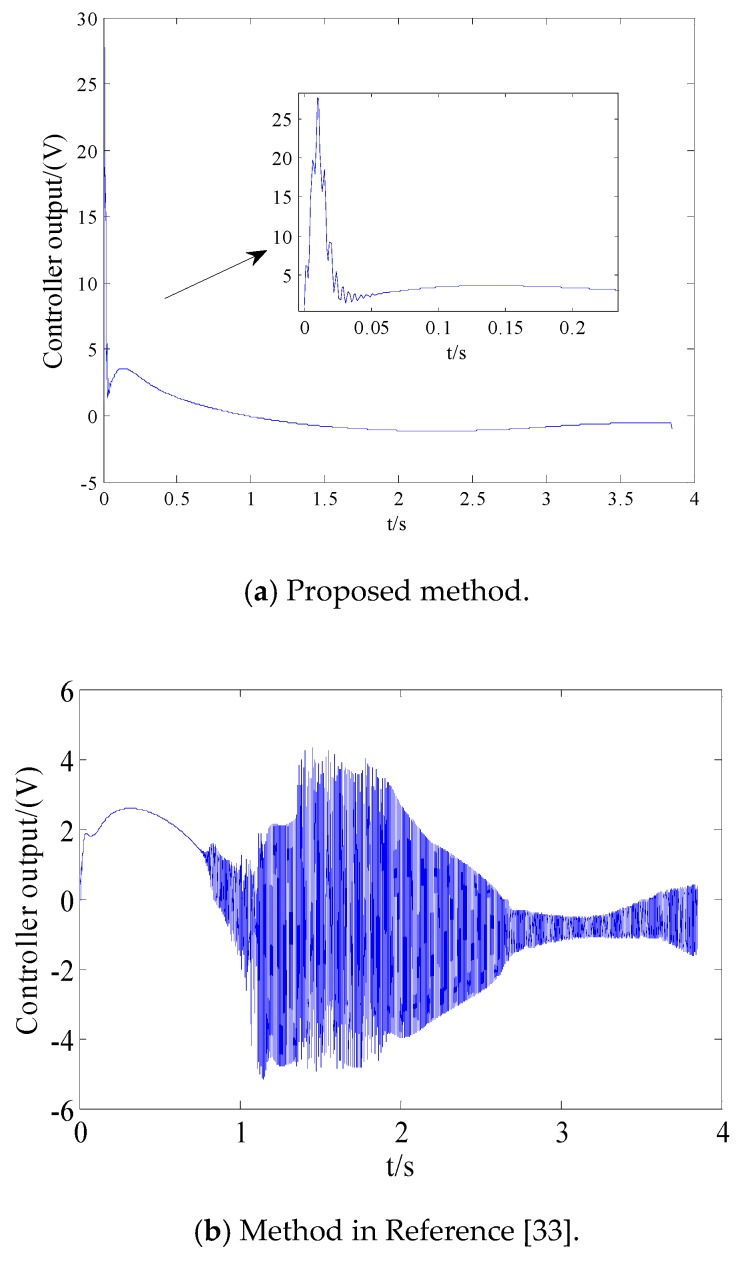
The controller output contrast.

From Figure 8a and Figure 9a, we can see that the convergence speed and tracking precision of the proposed method are better than the method in Reference [33]. Figure 10 shows the projectile disturbance generated in real time, and the figure shows that the disturbance is sharp. While Figure 11 shows the disturbance estimated by adaptive RBFNN. The designed adaptive RBFNN achieves precise compensation of disturbance, which will enhance the tracking ability. From Figure 8b and Figure 9b, we can see that a missile with the two mentioned methods hits the target in high precision. We can conclude that in this scenario, although the tracking performance of method in Reference [20] is worse than proposed method, its guidance precision is satisfying. The controller output contrast in Figure 12 shows that the controller output is smooth except for the initial big overshoot, while a sharp “chattering phenomenon” occurs in the sliding mode controller. We can conclude that the proposed method eliminates chattering of the controller output.

**Remark** **6.** *It should be noted that Figure 10 shows the disturbance generated by projectile motion* $\dot{\vartheta }$ *, while* Figure 11 *shows total disturbance* $D=\frac{L_{a}}{C_{m}k_{PWM}}(\frac{z_{2}}{z_{3}}(s+s_{c}+2k_{1})+(T_{turb}+(2-2k_{2}J_{L}){\dot{T}}_{turb}+\frac{C_{e}C_{m}\dot{\vartheta }}{L_{a}})/z_{3})$ *. The difference should be clarified.*

*Case 2.* In this case, we assume that an air-defense missile hits a plane target. The positions of missile and target are (0 m,10,000 m) and (8000 m,13,000 m), respectively; their initial speeds are 1000 m/s and 600 m/s, respectively, and the speed of target in *y*-axis direction is $V_{ty}=100\times \cos(0.2\pi t)(m/s)$. Thus, the LOSA is −0.358 rad, the initial servo system rotation angle is −0.34 rad, while the initial trajectory inclination angles are −30° and 0°, respectively.

Guidance law is chosen as the proportional navigation law, and the proportionality coefficient is K.

The order of HTD is chosen as 2, and the design parameters of HTD are chosen as follows: $R_{1}=200,R_{2}=20,R_{3}=5$, $a_{11}=10,a_{21}=10,a_{31}=10,a_{12}=10,a_{22}=10,a_{31}=10$; the control design parameters are $k_{1}=10,k_{2}=3,k_{3}=0.012$; parameters of adaptive RBFNN are chosen as $\gamma_{1}=1000,\gamma_{2}=3$, the number of node is 100, and the center of RBFNN is evenly spaced in c∈[−50,49], the width is chosen as b=15. Simulation step time is 0.001 s. 30% parameter perturbation of the servo system is introduced to verify the robustness.

**Remark** **7.** *The rules of control parameters are illustrated as follows. For a closed-loop control system, the primary purpose is to guarantee the stability based on the Lyapunov theorem of stability, while the stability interval of system falls in compact sets (Equation (46)). By choosing appropriate control parameters* $k_{1},k_{2},k_{3}$ *within the interval (Equation (45)), the system will be stable. Parameter* R *determines the response speed and estimation precision of HTD, its positive correlation, while* $a_{i}$ *determines the convergence characteristic of the* ith *term, its positive correlation, too. Parameters* $\gamma_{1},\gamma_{2},b$ *of RBFNN determines the convergence speed, besides,* $\gamma_{1},\gamma_{2}$ *determines the estimation precision directly. The center* c *is adjusted by the specific range of state variety, while the number of nodes depends on the complexity of estimation. The parameters of the control system have to be adjusted repeatedly.*

**Figure 13:**
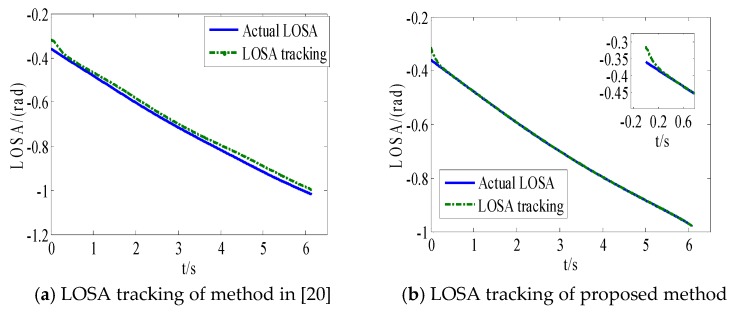
Line of sight angle (LOSA) tracking contrast.

It can be seen from Figure 13 that the proposed method converges quicker; besides, when there exists a 30% model parameters perturbation, the proposed method precisely tracks the reference, while the precision and robustness of the method in Reference [20] are worse.

**Figure 14:**
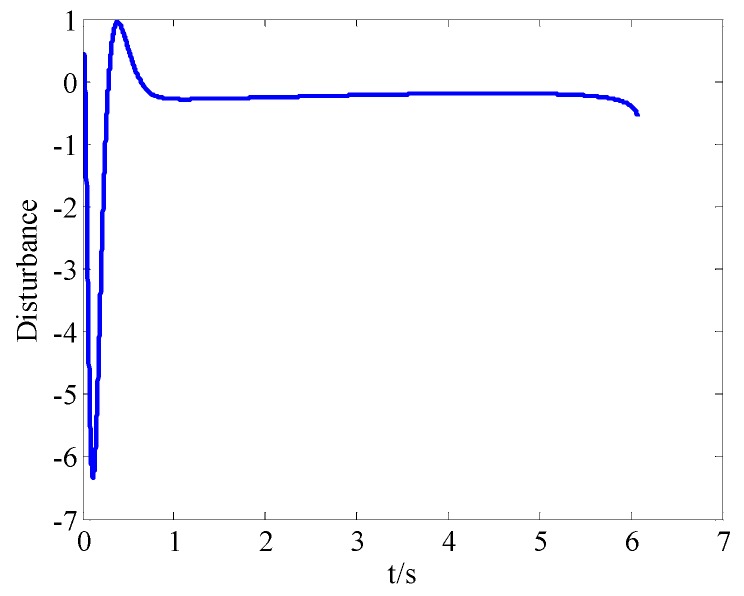
Projectile disturbance in real time.

**Figure 15:**
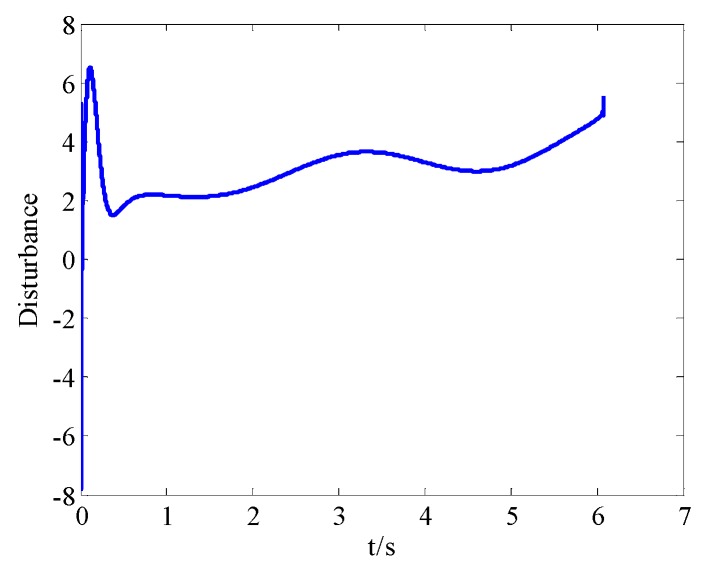
Total disturbance estimated by RBFNN.

**Figure 16:**
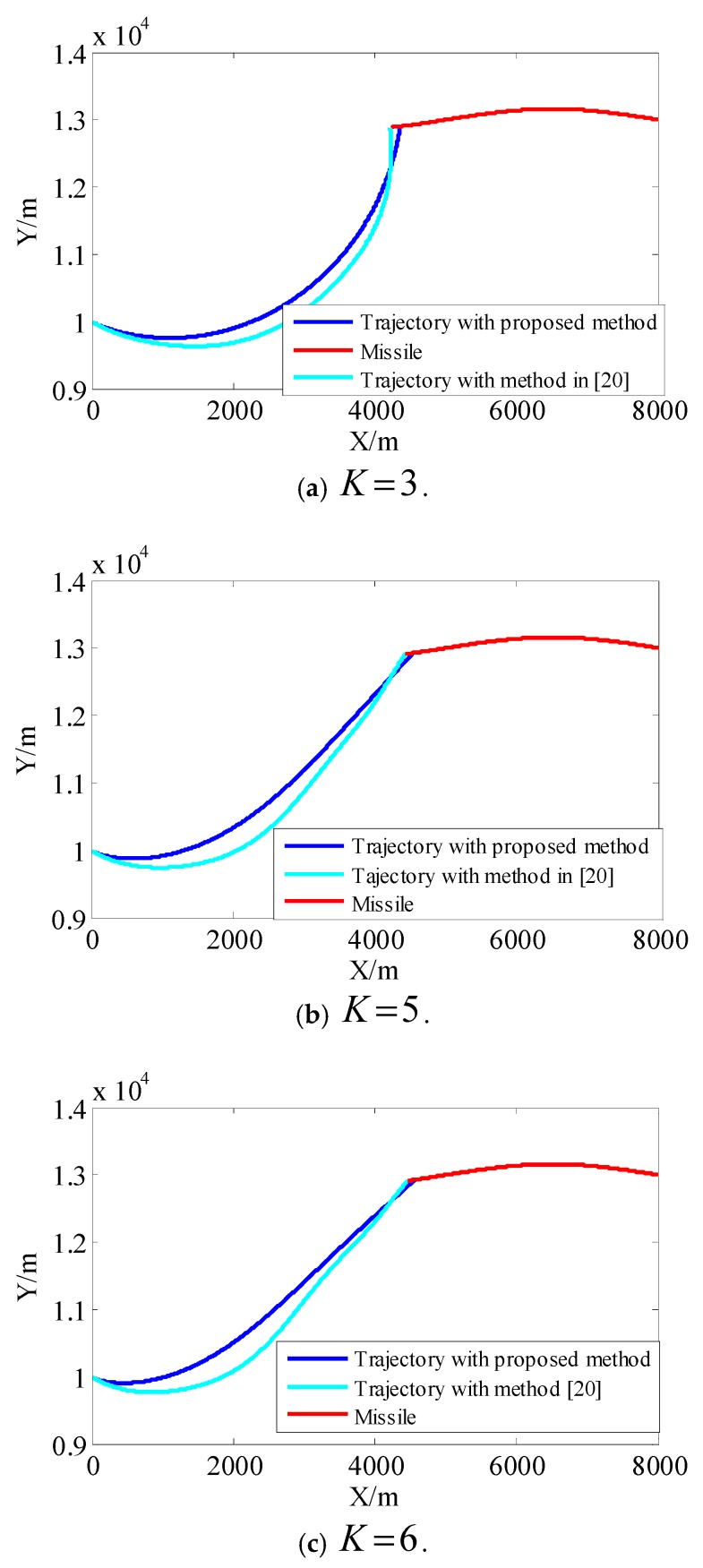
Contrast of trajectory with different guidance proportional coefficients.

Figure 14 shows the projectile generated in real-time, while Figure 15 shows total disturbance estimated by RBFNN. It can be observed from Figure 16 that a higher tracking performance of servo system advances hit time, and the trajectory is gentler.

To verify and demonstrate the advanced designed servo algorithm, a Monte Carlo simulation was carried out [34,35]. Through the Monte Carlo simulation, the effect of the improved servo algorithm is clearly shown through target-miss distance.

It can be seen from Table 2 that the designed method eliminates the miss distance in the considered scenarios.

## 5. Conclusions

In this paper, a backstepping sliding mode control based on an adaptive RBFNN is proposed to keep precise and stable tracking of seeker servo systems. A complete model of SGCS was built, and the algorithm design was carried out based on HTD. It is proved that all closed-loop system signals are semiglobally uniformly ultimately bounded. The designed method enhances the tracking robustness and precision in large scale, and solves problems of uncertain disturbance and parameters perturbation. A method in Reference [20] was compared with the proposed method, and the better performance of proposed method was proved through LOSA tracking contrasts. Besides that, Monte Carlo simulations showed that the proposed method enhances guidance precision.

## Figures and Tables

**Table 1:** Model Parameters.

Parameter	Value	Parameter	Value
$J_{L}$	$1.2\times 10^{-3}\;\mathrm{kg}\cdot m^{2}$	$C_{e}$	0.75
$C_{m}$	0.625 N⋅m/A	$k_{PWM}$	3.75
$L_{a}$	0.0062 H	$K_{W}$	0.05
$R_{a}$	5.1 Ω	$K_{N}$	1

**Table 2:** Monte Carlo simulation.

Method	Combat Scenario	Proportional Coefficient	Average Miss Distance/m
Proposed method	Low-altitude targets		0.338
	High-altitude targets	3	0.521
		5	0.785
		6	0.635
Method in [20]	Low-altitude targets		0.685
	High-altitude targets	3	0.946
		5	1.658
		6	1.885

## Data Availability

The data in this paper include two parts: one is model-parameter data and the other is simulation results generated by digital simulation. The model data are mostly derived from references, which have been remarked. The simulation results are derived from our original simulation program. All necessary data are presented in this paper.
